# Supplementary figures and images for: Detectable Lipidomes and Metabolomes by Different Plasma Exosome Isolation Methods in Healthy Controls and Patients with Advanced Prostate and Lung Cancer
Source: Int J Mol Sci. 2023 Jan 17;24(3):1830. doi: 10.3390/ijms24031830 (PMC9916336; doi:10.3390/ijms24031830)

Group    • Control    • Lung Cancer    • Prostate Cancer

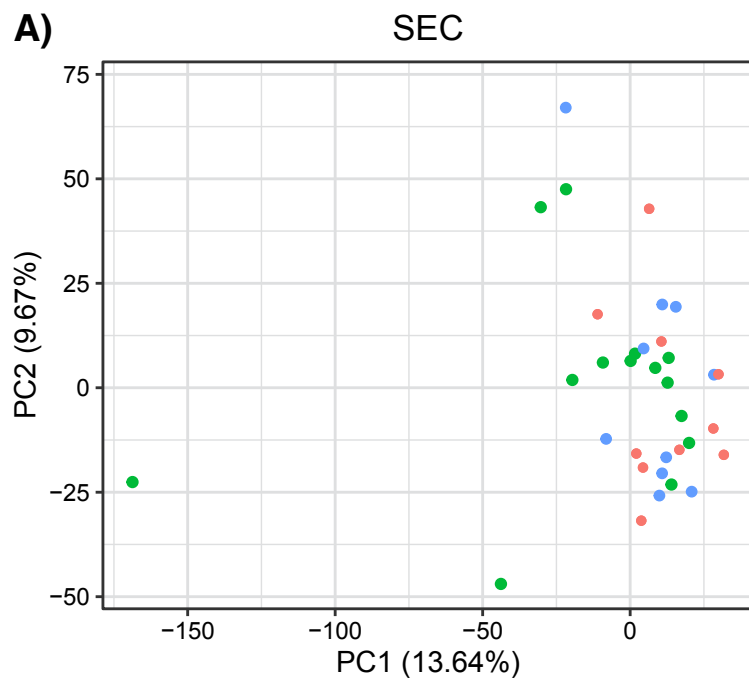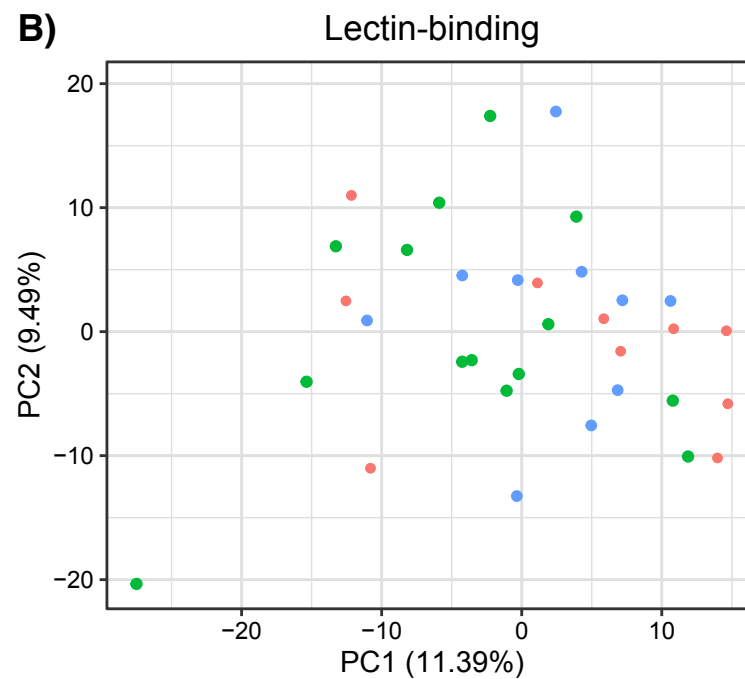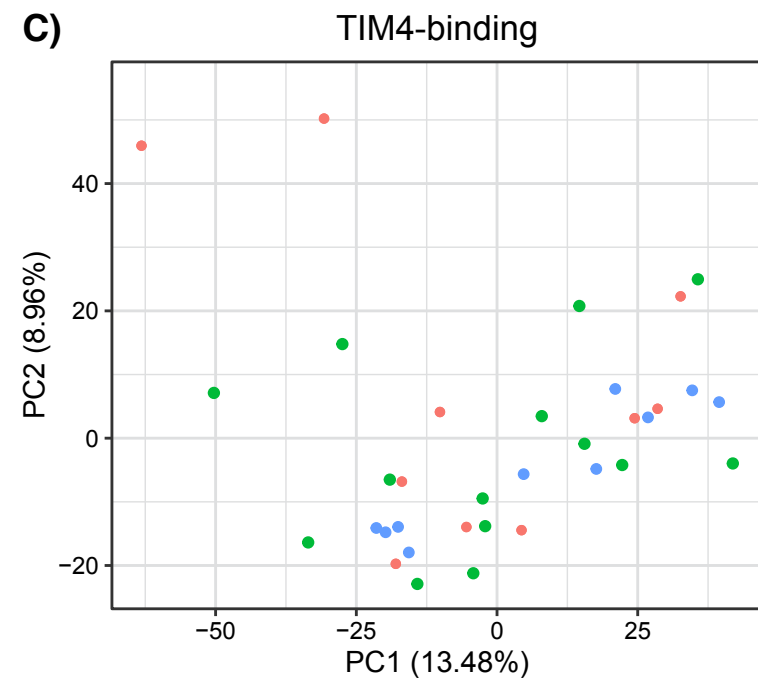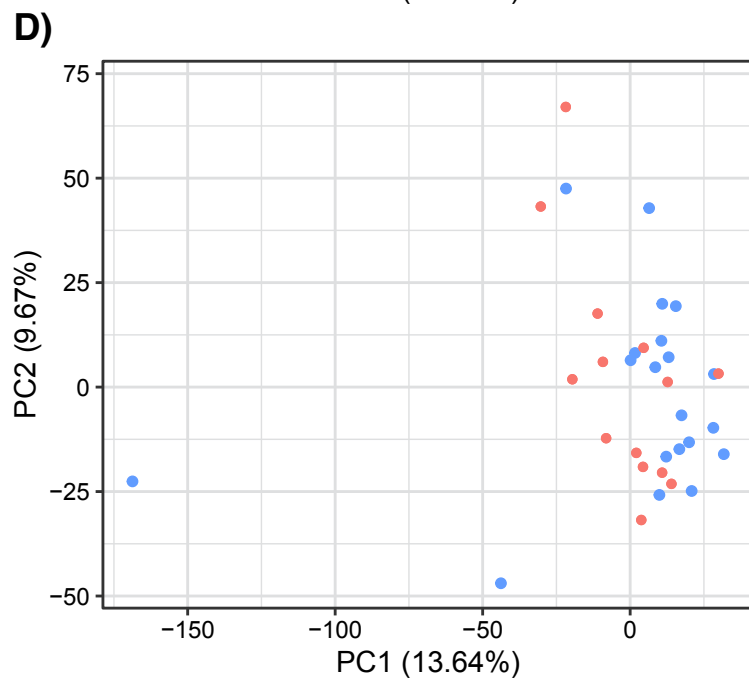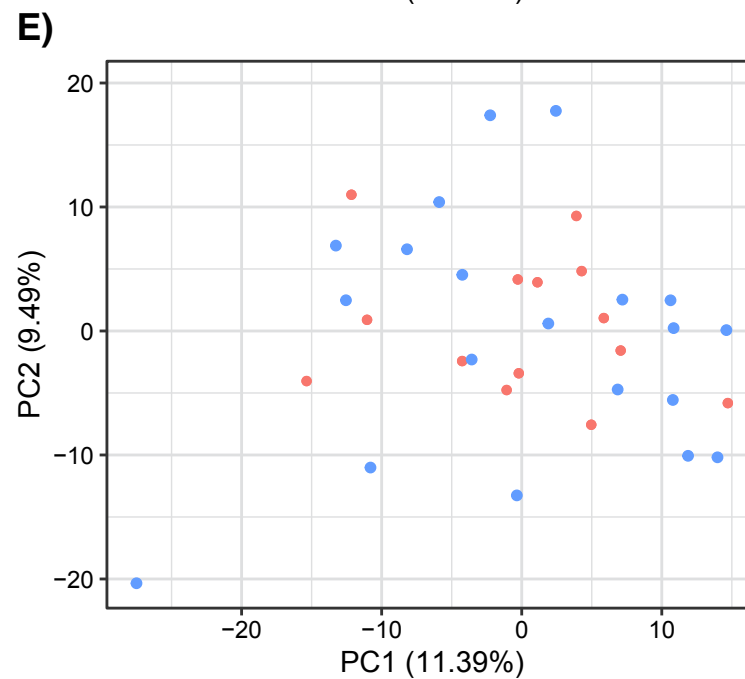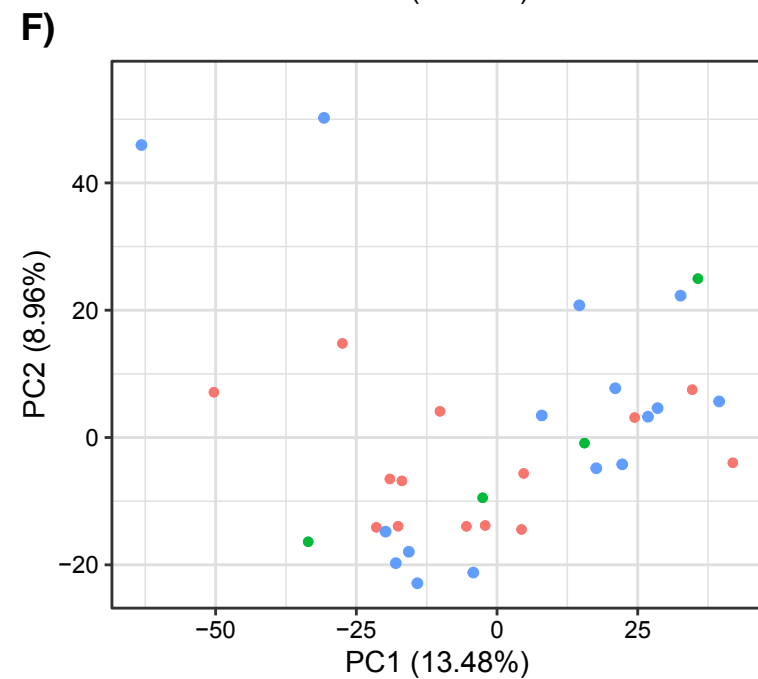

Batch    • 1    • 2    • 3

Supplement: Supplementary file 1 [file ijms-24-01830-s001.zip › SuppFigure1.pdf]

Group    ● Control    ● Lung Cancer    ● Prostate Cancer

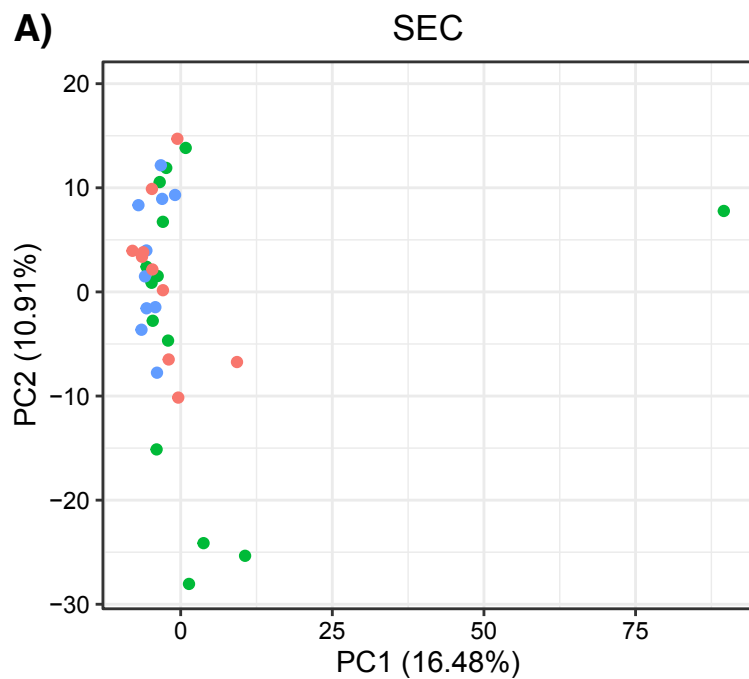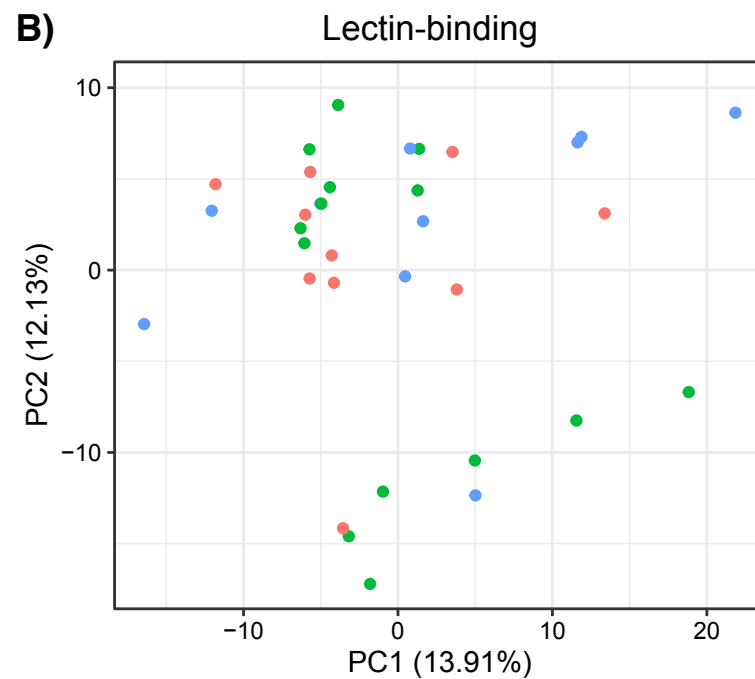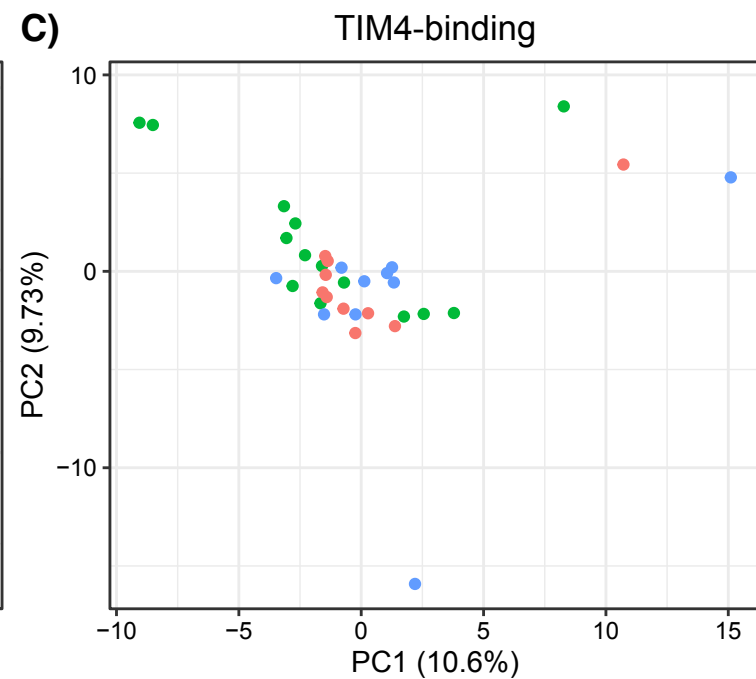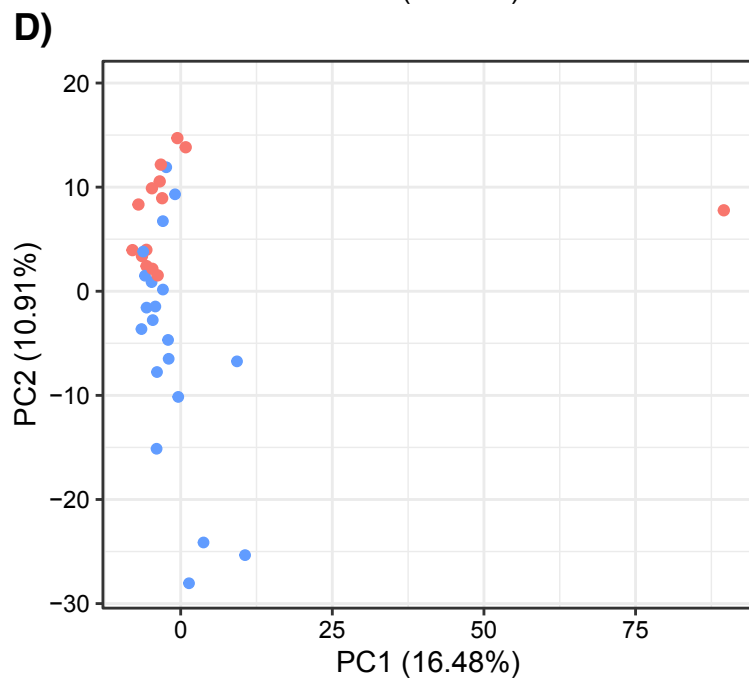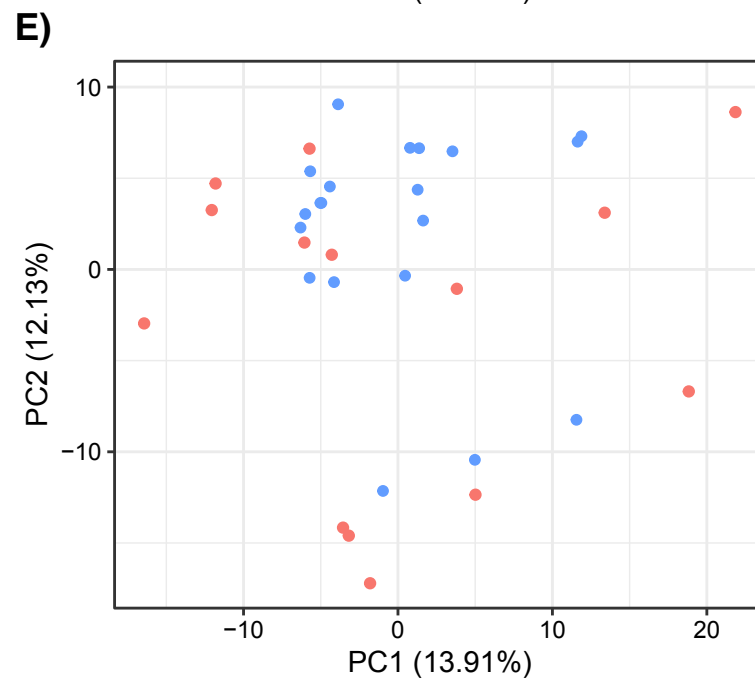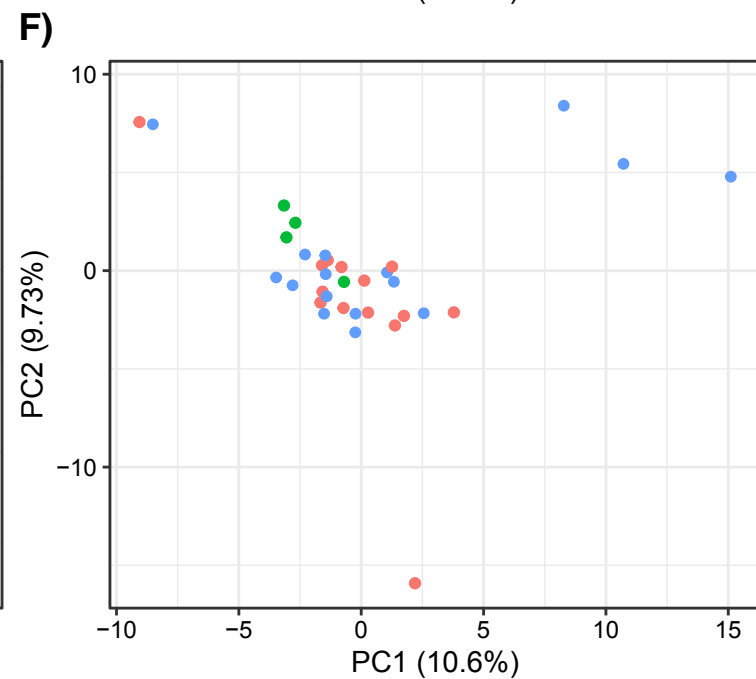

Batch    ● 1    ● 2    ● 3

Supplement: Supplementary file 1 [file ijms-24-01830-s001.zip › SuppFigure2.pdf]

Group    • Control    • Lung Cancer    • Prostate Cancer

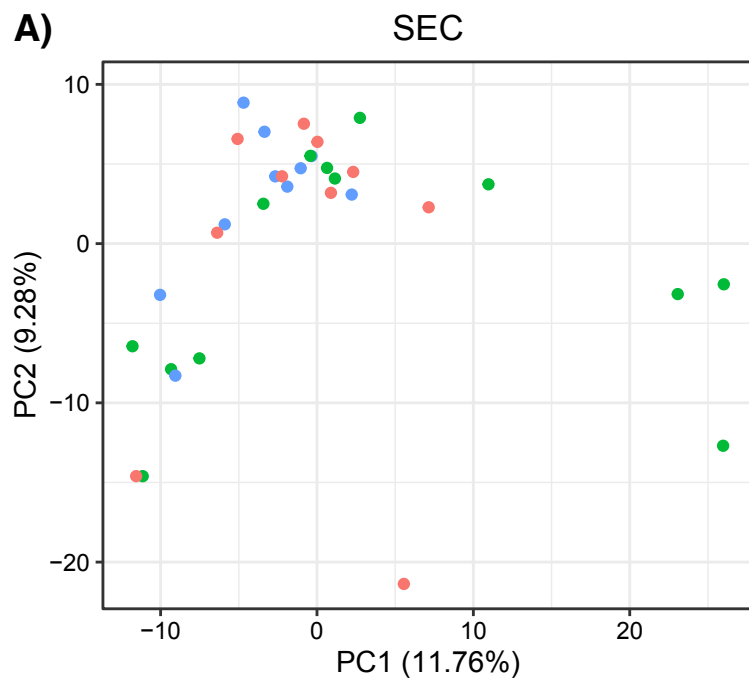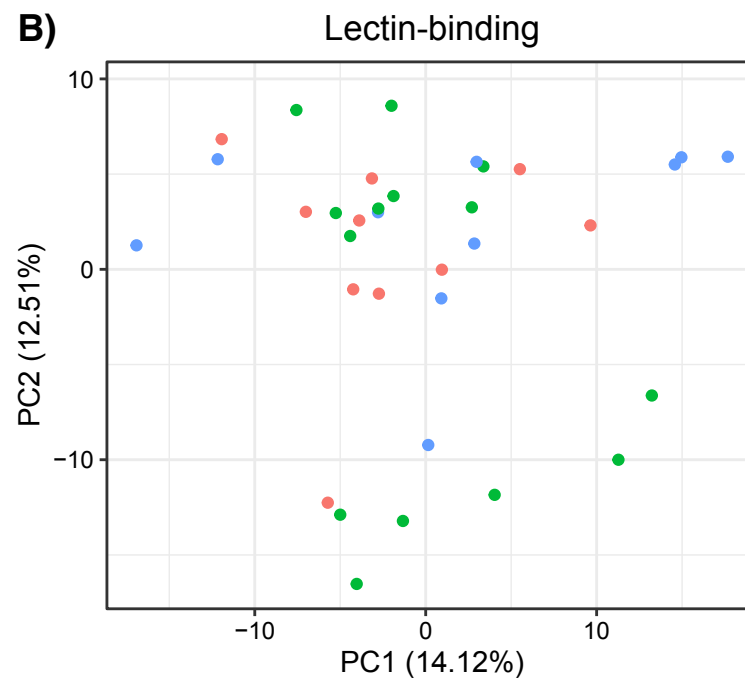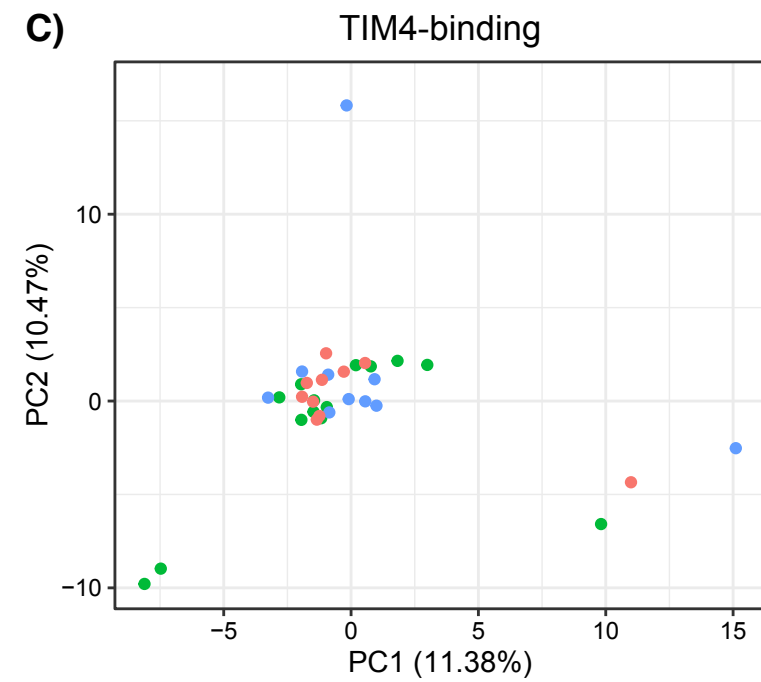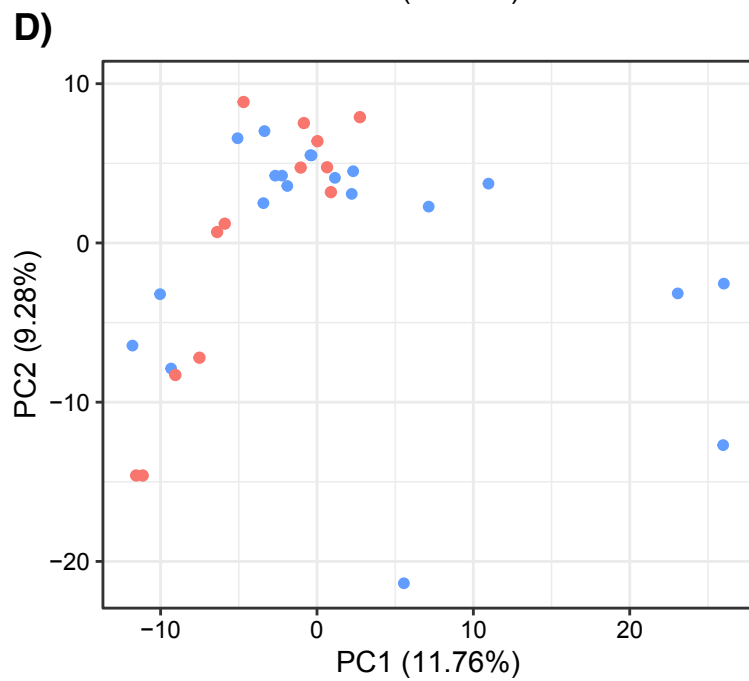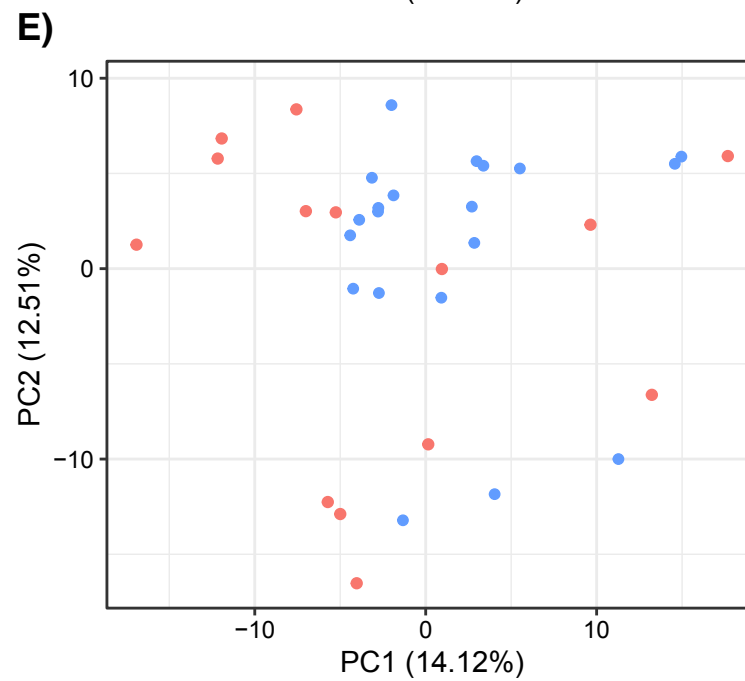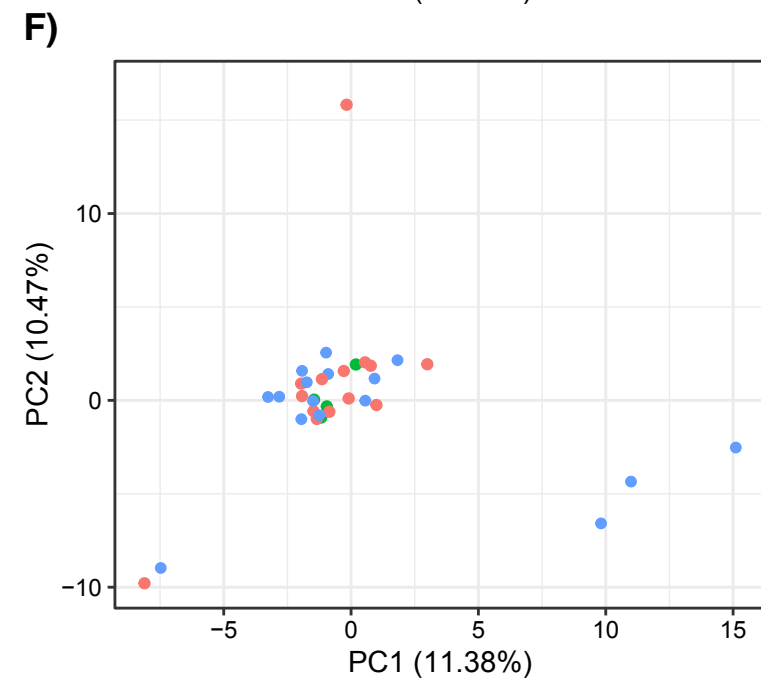

Batch    • 1    • 2    • 3

Supplement: Supplementary file 1 [file ijms-24-01830-s001.zip › SuppFigure3.pdf]

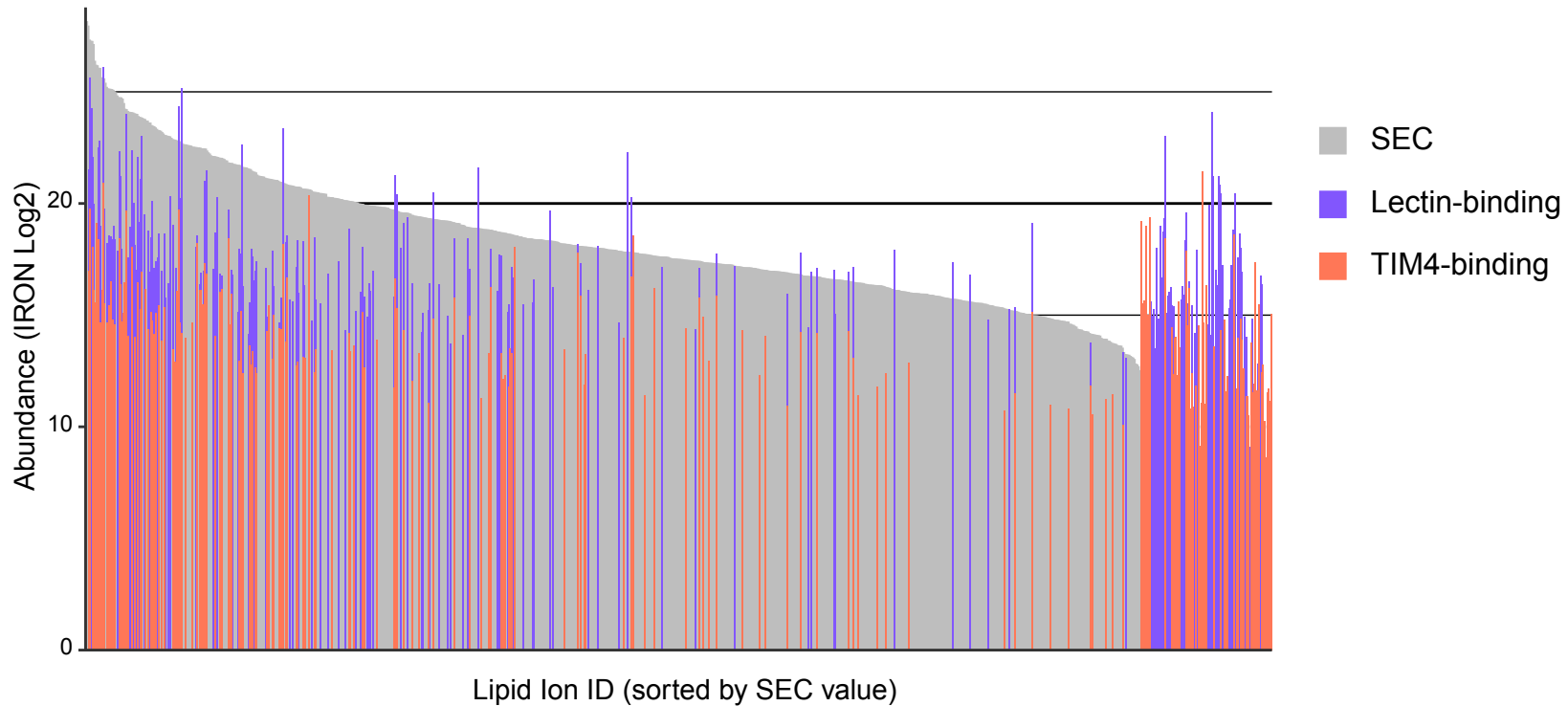

Supplement: Supplementary file 1 [file ijms-24-01830-s001.zip › SuppFigure4.pdf]

## A) Lipids

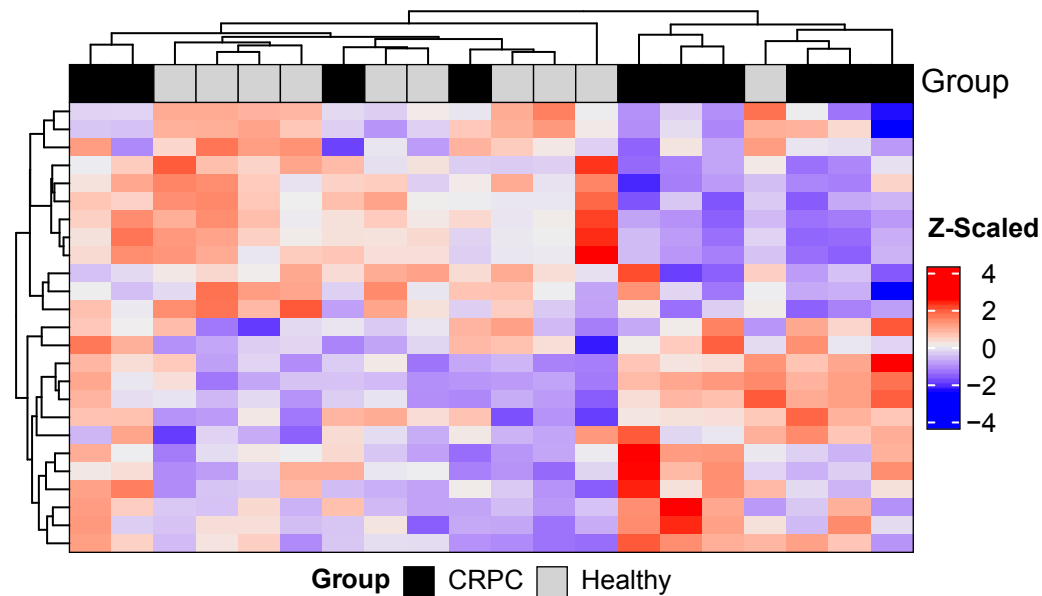

## B) Metabolites

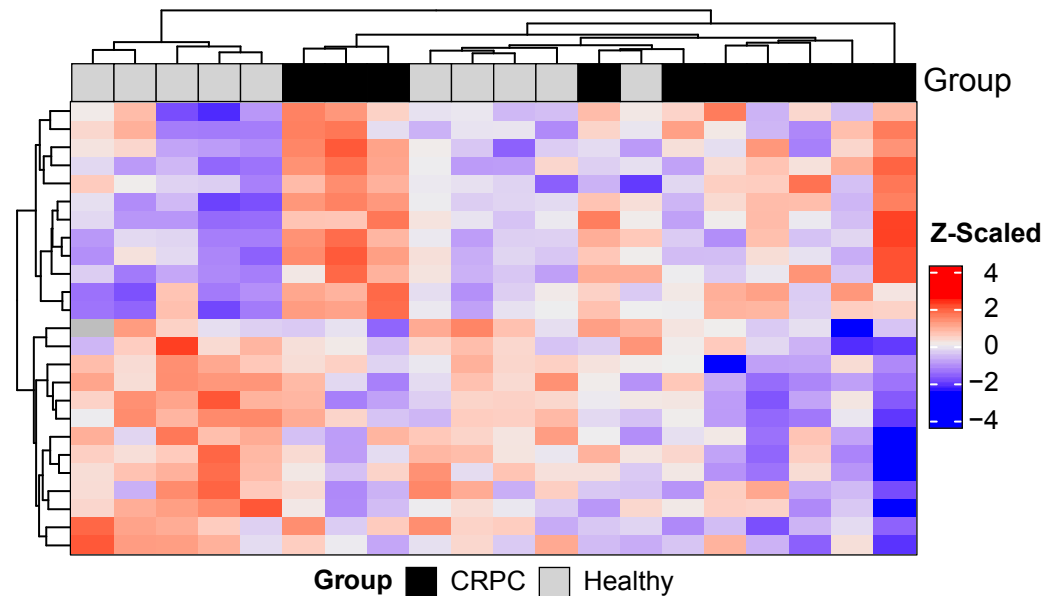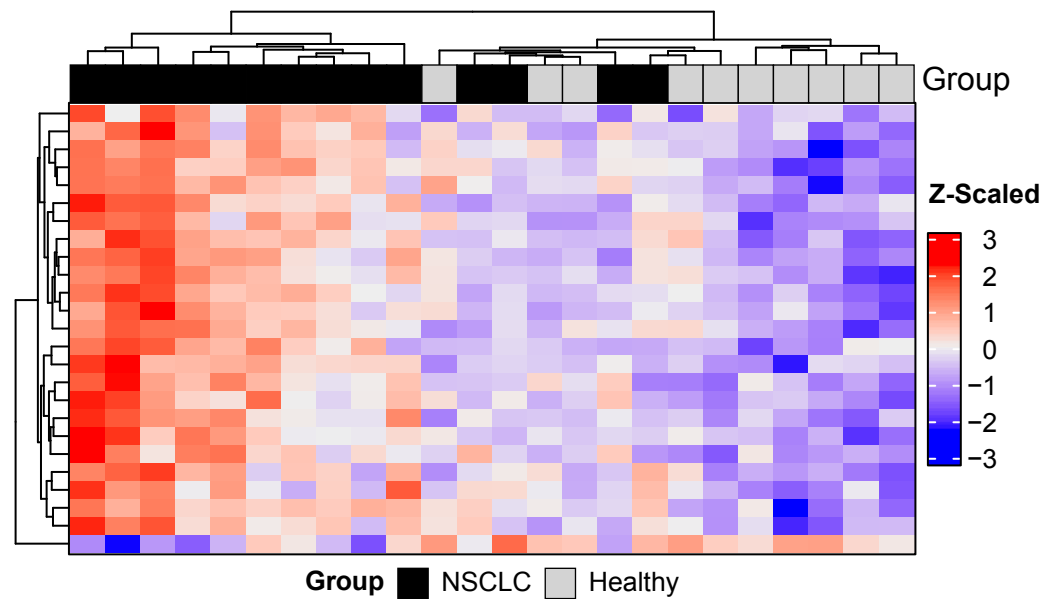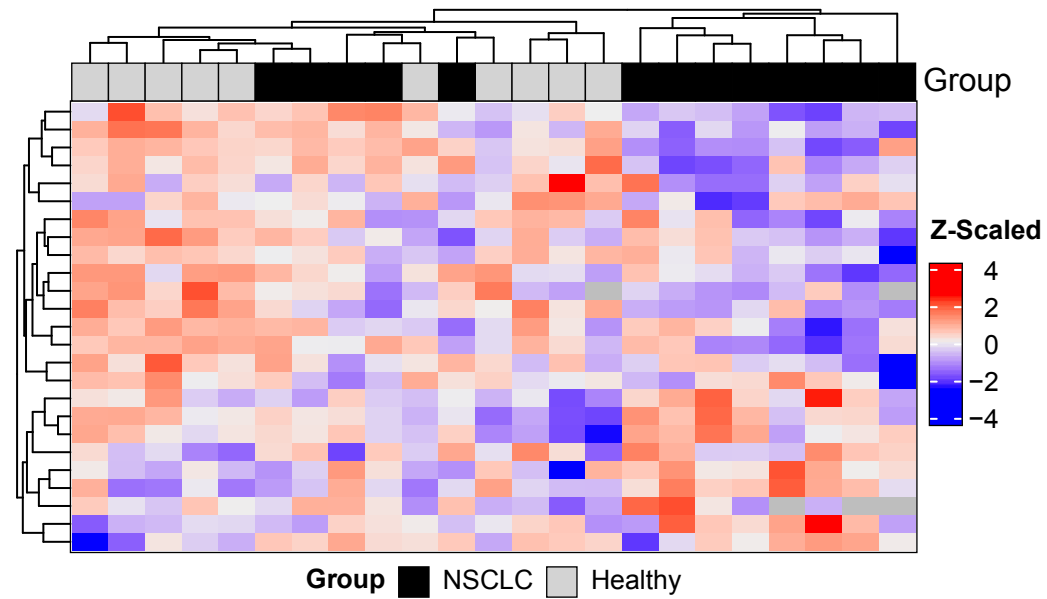

Supplement: Supplementary file 1 [file ijms-24-01830-s001.zip › SuppFigure5.pdf]

## A) Lipids

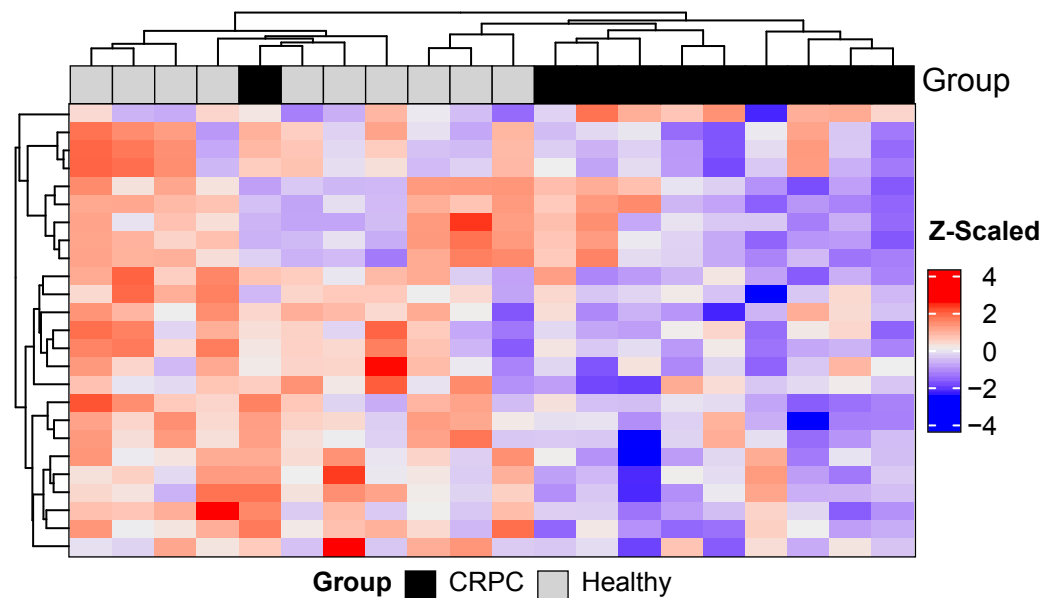

## B) Metabolites

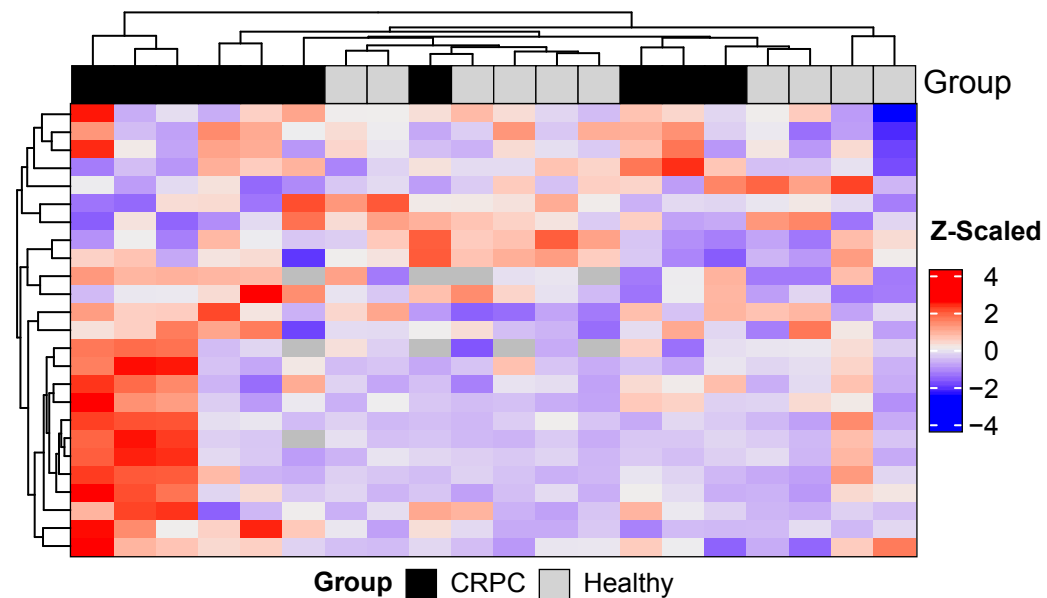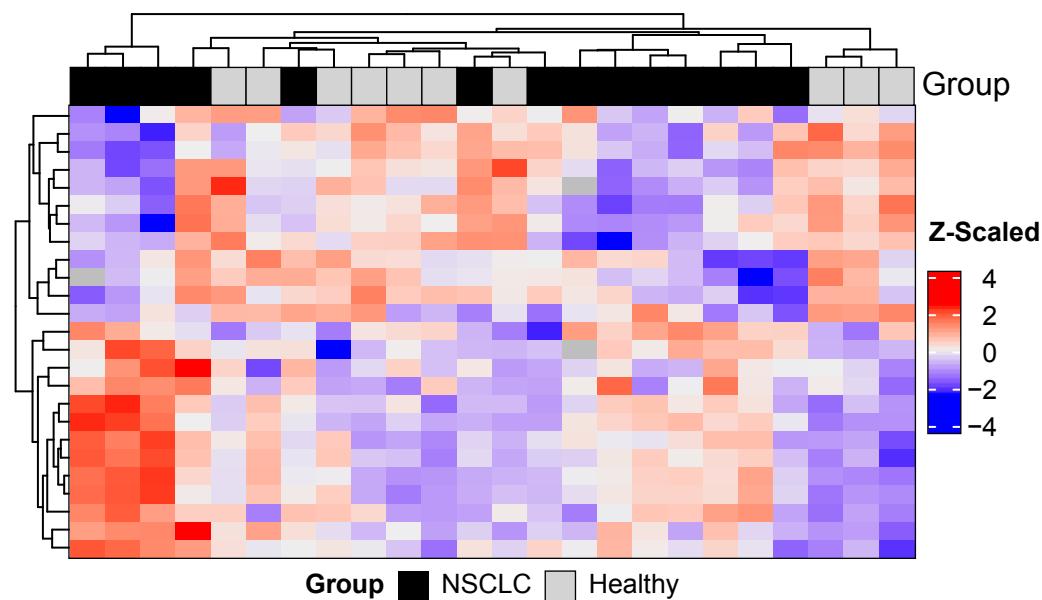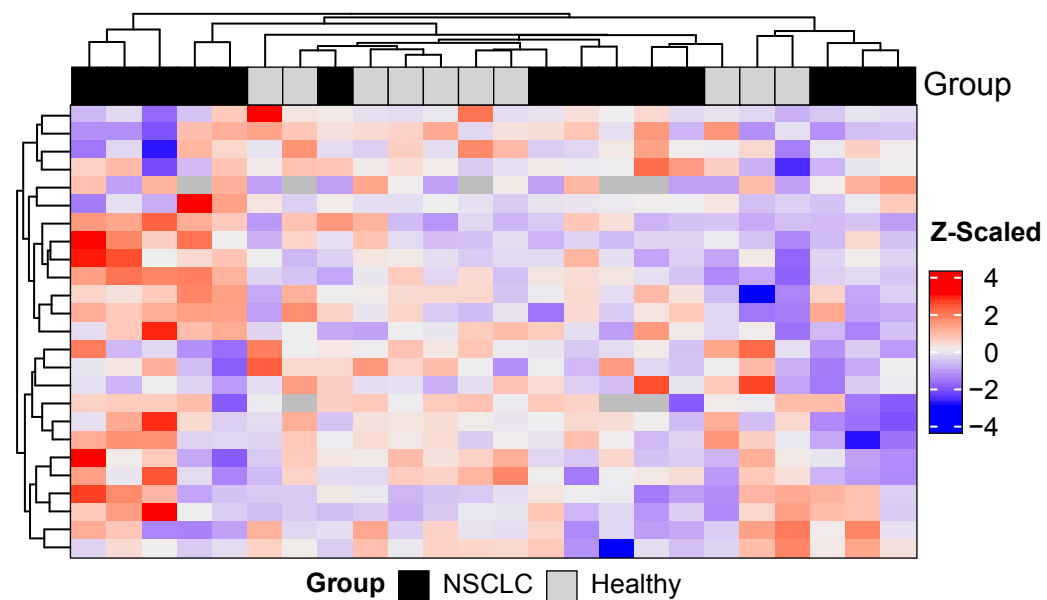

Supplement: Supplementary file 1 [file ijms-24-01830-s001.zip › SuppFigure6.pdf]

## A) Lipids

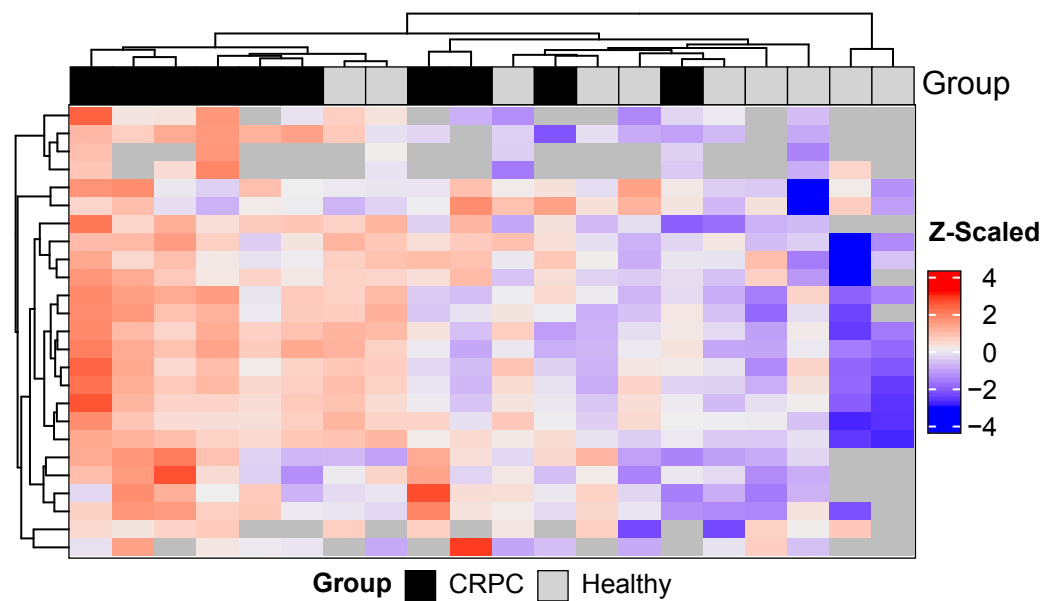

## B) Metabolites

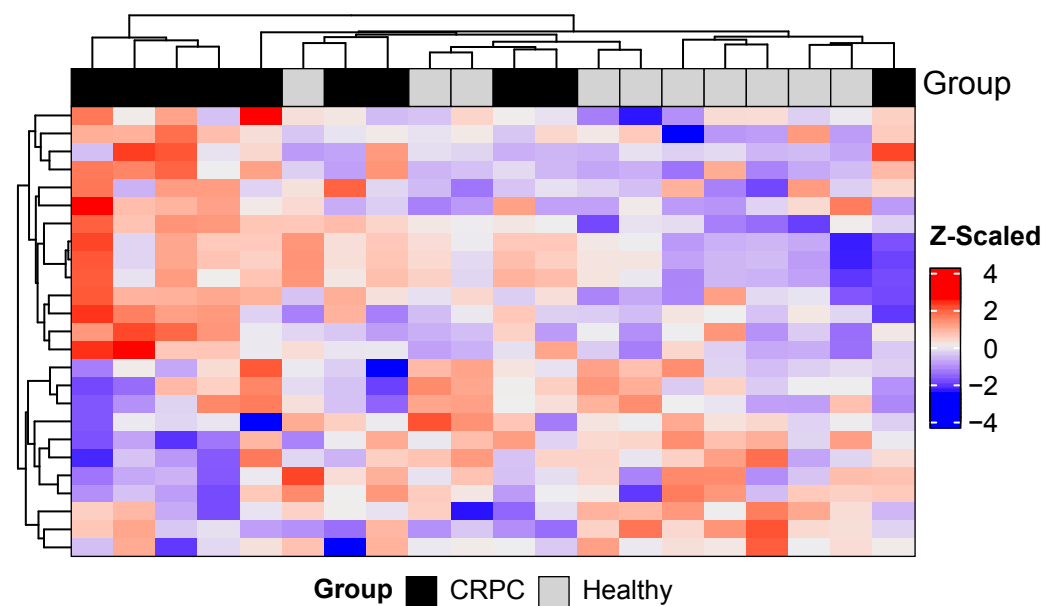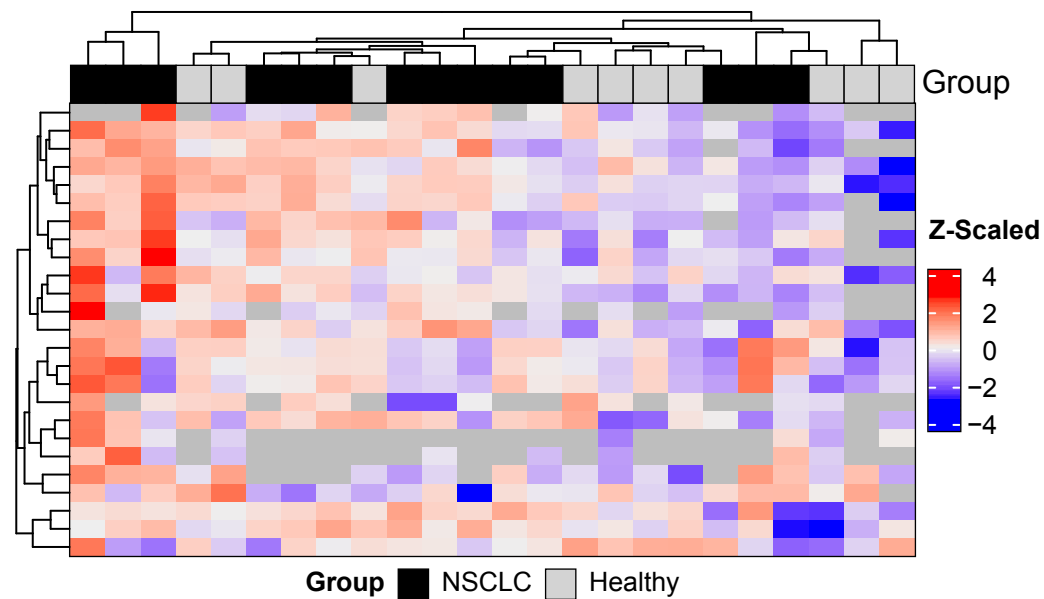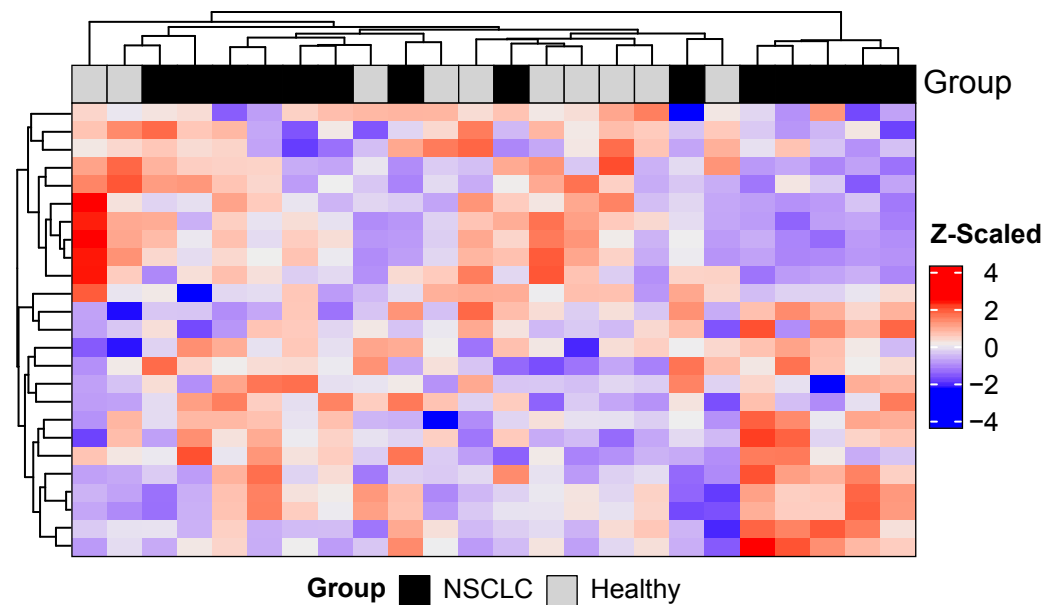

Supplement: Supplementary file 1 [file ijms-24-01830-s001.zip › SuppFigure7.pdf]

A)

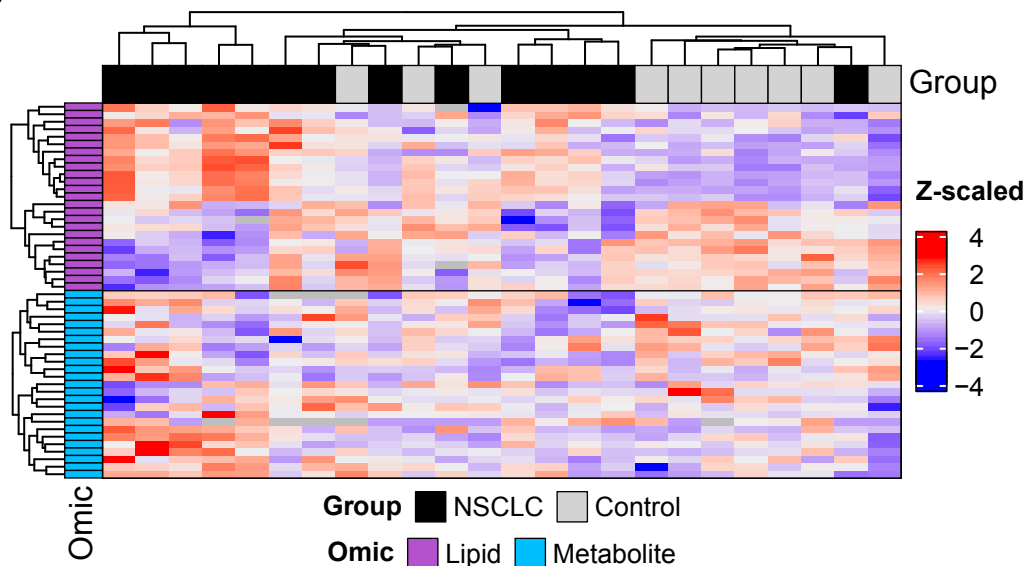

B)

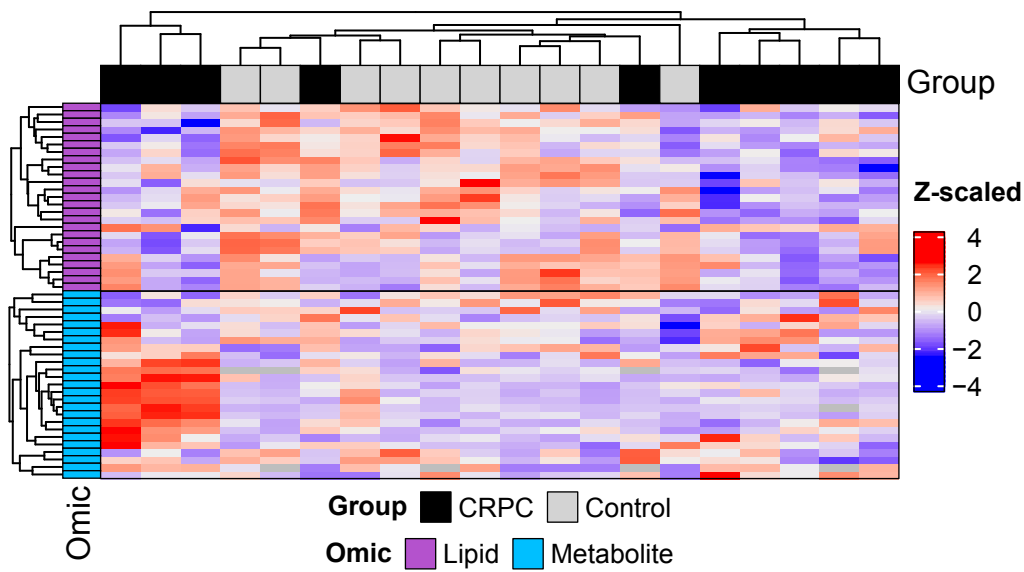

Supplement: Supplementary file 1 [file ijms-24-01830-s001.zip › SuppFigure8.pdf]

A)

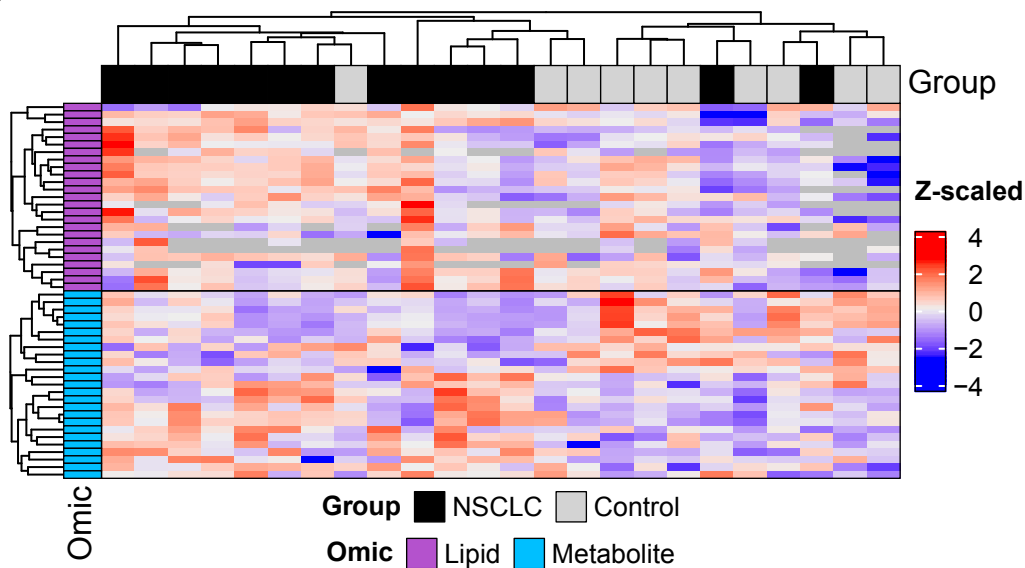

B)

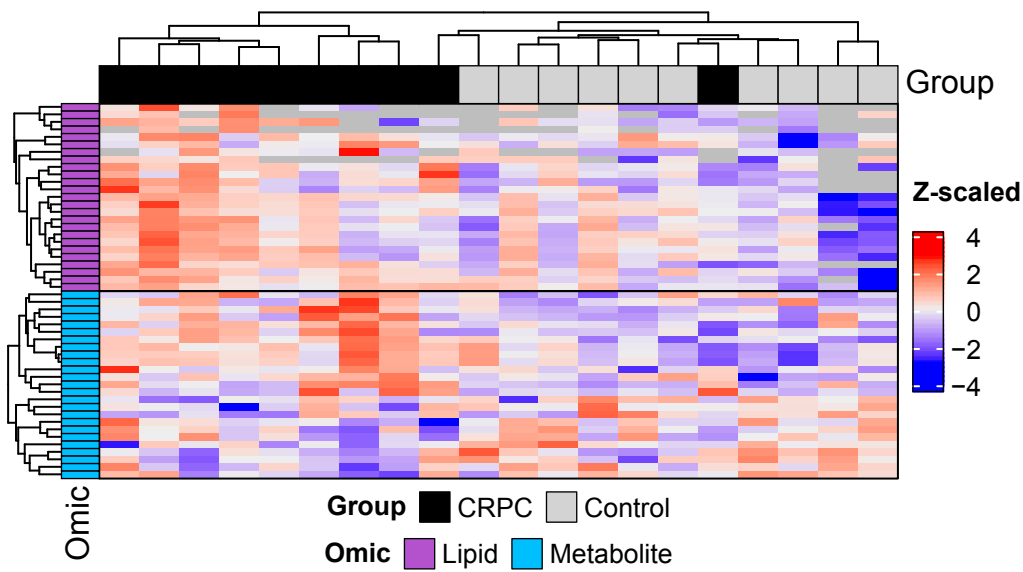

Supplement: Supplementary file 1 [file ijms-24-01830-s001.zip › SuppFigure9.pdf]
